# Compensatory swallowing methods in a patient with dysphagia due to lateral medullary syndrome—vacuum and prolonged swallowing: A case report

**DOI:** 10.1097/MD.0000000000028524

**Published:** 2022-01-07

**Authors:** Kenjiro Kunieda, Junya Sugiyama, Akiko Nomoto, Tomohisa Ohno, Takashi Shigematsu, Ichiro Fujishima

**Affiliations:** a Department of Neurology, Gifu University Graduate School of Medicine, Gifu, Japan; b Department of Rehabilitation Medicine, Hamamatsu City Rehabilitation Hospital, Hamamatsu, Japan; c Department of Dentistry, Hamamatsu City Rehabilitation Hospital, Hamamatsu, Japan.

**Keywords:** esophagus, high-resolution manometry, pressure, rehabilitation, Wallenberg

## Abstract

**Introduction:**

The nature of pharyngeal swallowing function during the course of recovery of dysphagia due to lateral medullary syndrome (LMS) is unclear. Vacuum swallowing is a compensatory swallowing method that improves the pharyngeal passage of a bolus by creating negative pressure during swallowing in the esophagus in patients with dysphagia due to LMS. We present a case involving a patient with dysphagia due to LMS who involuntarily acquired a swallowing method with prolonged and increased pharyngeal contraction and vacuum swallowing.

**Patient concerns:**

We report a unique case involving a 52-year-old patient with dysphagia due to LMS. His dysphagia was severe but improved gradually with swallowing rehabilitation. The patient involuntarily acquired a swallowing method with prolonged and increased pharyngeal contraction and vacuum swallowing.

**Diagnosis:**

The patient presented with dysphagia due to left LMS. A videofluoroscopic examination of swallowing revealed pharyngeal residue.

**Interventions:**

Forty-five days after the onset of the dysphagia, the swallowing pressure along the pharynx and esophagus was measured using high-resolution manometry.

**Outcomes:**

Vacuum swallowing was observed in six out of 19 swallows (32.5%). The velopharyngeal contractile integral (CI) and mesohypopharyngeal CI values increased during swallowing, reflecting prolonged and increased pharyngeal contraction. We named this swallowing method “prolonged swallowing.”

**Conclusion:**

The findings in this case indicate that vacuum and prolonged swallowing may be compensatory swallowing methods observed in individuals recovering from dysphagia due to LMS. Further research is needed to clarify the relationship between these swallowing methods and the pathophysiology, prognosis, and treatment of dysphagia in patients with LMS.

## 1 Introduction

Dysphagia is clinically significant in cases of lateral medullary syndrome (LMS) because it is related to aspiration pneumonitis, malnutrition, increased mortality, and prolonged hospital stay.^[1]^ The status of dysphagia in patients with LMS can range from very mild and transient to very severe and prolonged. With respect to prognosis, a majority of patients with LMS initially show severe dysphagia and require parenteral nutrition; however, such patients usually recover rapidly and return to oral food intake within a few months after the onset of dysphagia.^[1–6]^ In contrast, some patients with severe dysphagia require tube feeding and tracheostomy for several months or years, and may also require surgical treatment.^[1,7]^ With respect to patients with dysphagia, the nature of pharyngeal swallowing function in such patients during the recovery of dysphagia due to lateral medullary syndrome (LMS) is unclear.

Vacuum swallowing is a novel compensatory swallowing method that improves the pharyngeal passage of a bolus by creating negative pressure in the esophagus in patients who have dysphagia due to LMS.^[1,8,9]^ In this swallowing method, a bolus is sucked from the pharynx into the esophagus due to a pressure gradient despite weak pharyngeal contraction and impaired upper esophageal sphincter (UES) function.

Herein, we present a case involving a patient with dysphagia due to LMS who involuntarily acquired a swallowing method with prolonged and increased pharyngeal contraction and vacuum swallowing.

## 2 Patient information

The patient was a 52-year-old man who developed ataxia, alternating disturbance of pain and temperature sensation, dysarthria, and dysphagia. Diffusion-weighted imaging revealed high signal intensity (hyperintensity), indicating the occurrence of a left lateral medullary infarction that included the nucleus ambiguus. He was diagnosed as having left LMS. The patient was fed using a nasogastric tube. He exhibited dysphagia, and the severity of dysphagia according to the Food Intake LEVEL Scale (FILS) was level 3 (in patients with dysphagia of such severity, swallowing training using a small quantity of food is performed).^[10]^ Thirty-three days after the onset of dysphagia, he was admitted to our rehabilitation hospital for dysphagia treatment.

The patient had mild left ataxia, but could walk without a cane or walker. Sensory examination revealed reduced temperature and pinprick sensations on the left side of his face and right side of his extremity. He had dysphagia and mild hoarseness, and was expectorating saliva into a tissue. He underwent dysphagia rehabilitation once a day, including balloon catheter dilatation and head raising exercises, for impaired UES opening.

His dysphagia severity improved to FILS level 7 (in patients with dysphagia of such severity, easy-to-swallow food is orally ingested in three meals, and no alternative nutrition is provided). On day 45, a videoendoscopic examination of swallowing revealed pooling of saliva in the pyriform sinus and mild left vocal cord paralysis. A videofluoroscopic examination of the swallowing revealed a small amount of pharyngeal residue in the epiglottic vallecula and pyriform sinus. High-resolution manometry (HRM) revealed prolonged and increased constriction of the pharynx and impaired UES opening. Furthermore, the patient occasionally experienced negative pressure in the esophagus during swallowing.

Sixty-five days after the onset of dysphagia, HRM was performed for re-evaluation. The swallowing rehabilitation was continued, and the severity of dysphagia improved to FILS level 9 (there was no dietary restriction, and the patient ingested three meals orally; however, medical considerations were given). The patient was discharged 90 days after the occurrence of the stroke.

### 2.1 Manometric study

**Figure 1:**
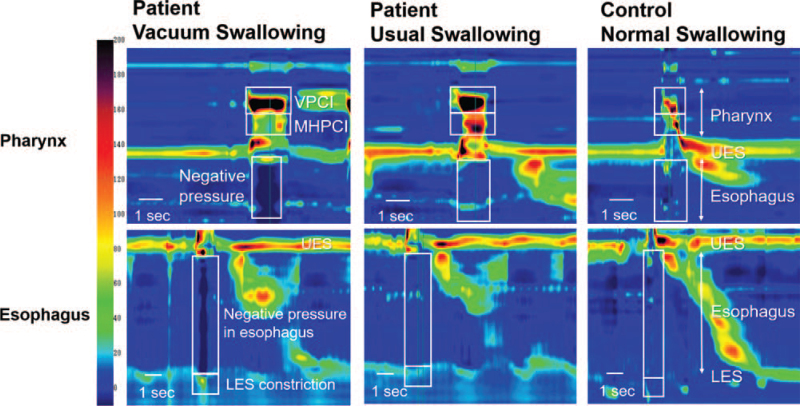
Pressure topography of the patient and a normal subject. Spatiotemporal plots for the patient in the present case and a normal subject (control) displaying vacuum swallowing (left), usual swallowing (middle), and normal swallowing (right). Y-axis, catheter position; X-axis, time. Pressure has been indicated using a color scale.

Pressure and timing data were extracted using the ManoScanTM software (Medtronic plc, Dublin, Ireland). Vacuum swallowing was defined as the simultaneous generation of strong negative esophageal pressure with swallowing. Values of several parameters, including the velopharyngeal contractile integral (VPCI), mesohypopharyngeal contractile integral, duration of UES relaxation, nadir UES pressure, minimum esophageal pressure (esophageal Pmin), and maximum lower esophageal sphincter (LES) pressure (LES Pmax), were recorded (Fig. 1). Contractile integrals were calculated using the following formula: amplitude × duration × length of muscular contractions ≥20 mm Hg.^[11,12]^ These integrals can act as useful indicators of pharyngeal swallowing disorders.

The mean and standard deviation (SD) values of each HRM parameter (on day 45) were estimated. Swallowing pressure in 19 swallows, including seven dry swallows, three swallows of thickened liquids for the pharyngeal phase, three dry swallows, three swallows of thickened liquids, and three swallows of sliced jellies for the esophageal phase, was evaluated. Vacuum swallowing was observed in six of the 19 swallows (32.5%), including two dry swallows, three thickened-liquid swallows, and one sliced-jelly swallow. A comparison of the means between the normal and vacuum swallowing was performed using the unpaired t-test. In the vacuum swallowing, the nadir UES pressure and esophageal Pmin were significantly lower than those in usual swallowing, and the LES Pmax was significantly higher than that in usual swallowing (Table 1). The VPCI, mesohypopharyngeal contractile integral, and nadir-UES-pressure values for the patient were greater than those for a normal subject,^[12]^ and the duration of UES relaxation in the patient was longer than that in a normal subject (control). The HRM-parameter values on day 65 were similar to day-45 values. The vacuum swallowing was observed in seven out of 24 swallows (29.1%).

**Table 1:** Summary of swallowing data.

	Vacuum swallowing	Usual swallowing	*P* value	Normal swallowing^[12]^
Pharynx	Three swallows	Seven swallows		
VPCI (mm Hg · s · cm)	836.9 ± 100.4	821.9 ± 21.8	.821	124.3 ± 50.3
MHPCI (mm Hg · s · cm)	432.4 ± 158.1	490.0 ± 48.8	.595	193.2 ± 34.1
UES relaxation duration (msec)	1193.3 ± 206.5	754.3 ± 286.9	.709	520.6 ± 60.0
Nadir UES pressure (mm Hg)	14.9 ± 10.7	19.7 ± 19.8	.046	−11.2 ± 6.7
Upper esophageal P_min_ (mm Hg)	−26.1 ± 9.2	−4.4 ± 1.4	.054	n/a
Esophagus	Three swallows	Six swallows		
Esophageal P_min_ (mm Hg)	−24.2 ± 3.1	−4.3 ± 2.2	<.001	n/a
LES P_max_ (mm Hg)	67.4 ± 11.0	24.5 ± 3.2	<.001	n/a

## 3 Discussion

To the best of our knowledge, this is the first report of a case involving a patient who had dysphagia due to LMS and involuntarily acquired vacuum and prolonged swallowing. These compensatory swallowing methods were revealed through the use of HRM, which was performed to derive precise information regarding pharyngeal and esophageal pressure.

It should be noted that the patient involuntarily acquired vacuum swallowing with the creation of negative pressure in the esophagus and increased LES pressure, which reflected the contraction of the diaphragm during swallowing. In a previously reported case, a patient voluntarily performed vacuum swallowing through the contraction of the respiratory and accessory muscles of respiration during swallowing^[1,8,13]^; this patient could also adjust the strength of the negative pressure in the esophagus by controlling the contraction of the respiratory muscles. In contrast, vacuum swallowing in the present case was performed involuntarily. Furthermore, the negative esophageal pressure during swallowing in the present case was not as strong as that in the patient in the previously reported case.

When we confirmed the presence of vacuum swallowing using HRM forty-five days after the onset of dysphagia, the bolus passed through the pharynx even with usual swallowing. The patient spontaneously acquired vacuum swallowing to compensate for impaired UES opening in the acute phase following the onset of LMS. As there is an improvement in swallowing function in such patients, the frequency of vacuum swallowing may gradually decrease. As vacuum swallowing can be reproduced using instructions,^[13]^ further research regarding the use of vacuum swallowing as a new swallowing maneuver in patients who have dysphagia due to LMS is needed.

Interestingly, involuntarily prolonged and increased constriction of the pharynx was observed in the present case. Pharyngeal contractile integrals are measures of the “vigor” of pharyngeal contractility, including prolonged swallowing time. In the present case, the remarkable increase in the VPCI reflected the increased and prolonged contraction of the velopharynx during swallowing. This might be similar to the Mendelsohn maneuver, which requires the palate and larynx to be held forcefully while swallowing.^[14,15]^ These findings are empirically observed in patients using videofluoroscopy, specifically in the recovery process of bulbar-type dysphagia due to LMS. We named this swallowing method “prolonged swallowing,” which refers to prolonged pharyngeal contraction time.

The central pattern generator for swallowing, nucleus ambiguus, and nucleus tractus solitarius, located in the medulla, control the oropharyngeal phase of swallowing.^[1,2,4,5,11]^ An LMS lesion involving the central pattern generator, nucleus ambiguous, and nucleus tractus solitarus could cause decreased pharyngeal contractility and impaired UES function.^[1,11]^ Vacuum and prolonged swallowing might be compensatory swallowing maneuvers that are used due to weak pharyngeal contractility and impaired UES opening in patients who have dysphagia due to of the LMS.

A majority of patients with LMS initially exhibit severe dysphagia; however, they often recover within few months of the onset of LMS. Several patients who progress to oral food intake may acquire these swallowing methods. It is not known how frequently these swallowing methods are observed in such patients and how long they persist in the recovery process that follows the onset of LMS. In the previously reported case, the subject performed vacuum swallowing consciously.^[8]^ In contrast, in the present case, vacuum swallowing was acquired and occurred involuntarily. Additional studies are necessary to determine whether these swallowing methods can be applied in clinical practice as a new therapeutic approach to improve swallowing.

In conclusion, patients with dysphagia due to LMS may utilize vacuum and prolonged swallowing as compensatory swallowing methods due to abnormal pharyngeal contraction and impaired UES opening during the recovery process following the onset of dysphagia and LMS. Further research is needed to clarify the relationship between these swallowing methods and the pathophysiology, prognosis, and treatment of dysphagia in patients with LMS.

## Acknowledgments

The authors thank Editage for English language editing.

## Author contributions

KK and IF: developed the study concept and design. JS and AN: acquired the data. KK and TO: analyzed and interpreted the data. KK: drafted the manuscript. All six authors: approved the final version to be submitted.

**Conceptualization:** Kenjiro Kunieda.

**Data curation:** Kenjiro Kunieda, Junya Sugiyama, Akiko Nomoto, Ichiro Fujishima.

**Formal analysis:** Tomohisa Ohno.

**Investigation:** Kenjiro Kunieda, Junya Sugiyama, Akiko Nomoto.

**Methodology:** Kenjiro Kunieda.

**Project administration:** Kenjiro Kunieda.

**Supervision:** Tomohisa Ohno, Takashi Shigematsu, Ichiro Fujishima.

**Writing – original draft:** Kenjiro Kunieda.

**Writing – review & editing:** Kenjiro Kunieda, Tomohisa Ohno, Takashi Shigematsu, Ichiro Fujishima.

## References

[1] Jang SH Kim MS. Dysphagia in lateral medullary syndrome: a narrative review. Dysphagia 2021;36:329–38. 32654058 10.1007/s00455-020-10158-3

[2] Kim JS. Pure lateral medullary infarction: clinical-radiological correlation of 130 acute, consecutive patients. Brain 2003;126:1864–72. 12805095 10.1093/brain/awg169

[3] Crary MA. A direct intervention program for chronic neurogenic dysphagia secondary to brainstem stroke. Dysphagia 1995;10:06–18. 10.1007/BF00261273 7859537

[4] Meng NH Wang TG Lien IN. Dysphagia in patients with brainstem stroke: incidence and outcome. Am J Phys Med Rehabil 2000;79:170–5. 10744192 10.1097/00002060-200003000-00010

[5] Kim H Chung CS Lee KH Robbins J. Aspiration subsequent to a pure medullary infarction: lesion sites, clinical variables, and outcome. Arch Neurol 2000;57:478–83. 10768620 10.1001/archneur.57.4.478

[6] Kim HJ Lee HJ Park JW. Clinical course and outcome in patients with severe dysphagia after lateral medullary syndrome. Ther Adv Neurol Disord 2018;11: 1756286418759864. 10.1177/1756286418759864 PMC5833167 29511384

[7] Montgomery WW. Surgery to prevent aspiration. Arch Otolaryngol 1975;101:679–82. 1200909 10.1001/archotol.1975.00780400037010

[8] Kunieda K Kubo S Fujishima I. New swallowing method to improve pharyngeal passage of a bolus by creating negative pressure in the esophagus-vacuum swallowing. Am J Phys Med Rehabil 2018;97:e81–4. 29194048 10.1097/PHM.0000000000000872 PMC6092101

[9] Jones CA Colletti CM Ding MC. Post-stroke dysphagia: recent insights and unanswered questions. Curr Neurol Neurosci Rep 2020;20:61. 33136216 10.1007/s11910-020-01081-z PMC7604228

[10] Kunieda K Ohno T Fujishima I Hojo K Morita T. Reliability and validity of a tool to measure the severity of dysphagia: the Food Intake LEVEL Scale. J Pain Symptom Manage 2013;46:201–6. 23159683 10.1016/j.jpainsymman.2012.07.020

[11] Kunieda K Sugi T Ohno T. Incoordination during the pharyngeal phase in severe dysphagia due to lateral medullary syndrome. Clin Case Rep 2021;9:1728–31. 33768924 10.1002/ccr3.3890 PMC7981674

[12] Kunieda K Fujishima I Wakabayashi H. Relationship between tongue pressure and pharyngeal function assessed using high-resolution manometry in older dysphagia patients with sarcopenia: A pilot study. Dysphagia 2021;36:33–40. 32140906 10.1007/s00455-020-10095-1

[13] Kunieda K Kubo S Fujishima I. A new swallowing method to improve pharyngeal passage by creating negative pressure in the esophagus-vacuum swallowing: reproduction in normal subjects. Deglutition 2018;7:224–30. 10.1097/PHM.0000000000000872 PMC6092101 29194048

[14] Hoffman MR Mielens JD Ciucci MR Jones CA Jiang JJ McCulloch TM. High-resolution manometry of pharyngeal swallow pressure events associated with effortful swallow and the Mendelsohn maneuver. Dysphagia 2012;27:418–26. 22215280 10.1007/s00455-011-9385-6 PMC3717357

[15] Ding R Larson CR Logemann JA Rademaker AW. Surface electromyographic and electroglottographic studies in normal subjects under two swallow conditions: normal and during the Mendelsohn maneuver. Dysphagia 2002;17:01–12. 10.1007/s00455-001-0095-3 11820381

